# Diseases of the Maxillary Sinus or Antrum

**Published:** 1889-03

**Authors:** Louis Jordan

**Affiliations:** Delphi, Ind.


ARTICLE VI.

### DISEASES OF THE MAXILLARY SINUS OR ANTRUM.

DR. LOUIS JORDAN, DELPHI, IND.

It was not till the knowledge of anatomy had made considerable progress that the existence of this cavity was known. The credit of a correct description belongs to Nathaniel Highmore; hence its name, "Antrum Highmorianum."

This cavity is subject to some of the most formidable and dangerous diseases the medical or surgical practitioner is called on to treat. Diseases are sometimes here met which only ends in death. An old gentleman, a farmer, in the seventies, had been afflicted for many years with fic-dolerean, and, getting no relief through medical aid, came to consult me. I told him I would try a remedy, but was doubtful of its doing him any good. It was a preparation of salammoniac. It had but little effect, and he lingered for two or three years more, afflicted with most excruciating pain, till death relieved him.

It is not all the diseases of the antrum that are of so dangerous a character; some are simple and easily cured. Those that are recorded as the least dangerous, and would yield most readily to treatment in the earlier stages of the disease, often, if neglected or improperly treated, assume a new and aggravated form, and bid defiance to the skill of the physician and surgeon.

The form which the disease puts on is determined by the state of the constitution or the sequence of some disease badly treated. The cause, which in one person would give rise to a simple inflammation of the lining membrane, or mucous engorgement of the sinus, would in another produce an ill-conditioned ulcer, fungous hematodes or osteosarcoma. In many cases of disease of the maxillary sinus, the danger to be apprehended results more from neglect than any necessarily fatal character of the malady.

In forming a prognosis, the circumstances to be considered are the state of the health, the progress of the affection, and the nature of the injury inflicted by it on the surrounding tissues. In young or middle-aged persons of good constitutions, a morbid action may exist in the antrum for years without exhibiting the least alarming symptom. Where the constitution is less healthy, it may rapidly change into a form of disease so malignant as to endanger the life of the patient. The qualities of the secretions are changed, and a nauseating, fetid odor is thrown out. In many instances it is almost insufferable, and when prevented from escaping through the natural channel into the nose, they discharge through one formed by art, or break through the cheek, alveolar border or palatine arch. It may exist for weeks or months before its presence is suspected.

Man is not the only animal affected with disease of the antrum Dr. William Cook says a disease prevailed among cattle which caused them to go mad, and it was believed they were bitten by a mad dog. Having a desire to ascertain the seat and nature of the malady, he examined the head of one that died of it. He first directed his research to the brain, but no abnormal appearance were observable; he then laid open the superior maxillary cavity and found it filled with fœtid matter.

The extremity of the root of a tooth may perforate the sinus, causing inflammation of the lining membrane, and when matter is thus formed in the alveolus of the superior molars, instead of forcing its way through the socket of the tooth, it will escape into that cavity, mixing with its secretions, and pass out through the nasal opening.

Inflammation and ulceration of the pituitary membrane of the nose may extend to the maxillary sinus, but it is seldom that both are affected at the same time.

Diseases of the nasal fosse, as catarrhal affections, may occasionally give rise to the morbid condition of the maxillary sinus. Concealed as it is, with the essential part of the superior maxilla, and being connected with the nose by a very small opening, it would seem beyond the reach of most causes to which diseases of the nasal fosse are attributed.

The opening of this cavity may sometimes be closed by a disease in the nose; and, if so, it is followed by a mucus engorgement of the sinus, inflammation of its lining membrane, and dissention of its osseous walls, and sometimes by a more complicated disease, as polypus.

The pain occasioned by a diseased tooth is often severe, and the inflammation excited by them in the alveoli dental periostea and gums frequently extends to the whole of one side of the face. Some writers attribute these affections to the morbid condition of the teeth and alveoli, other diseases, blows on the face, and exposure to cold. The pituitary membrane of the antrum is shielded from the irritating agents to which it is exposed in the nasal fosse and other cavities. It would seldom be affected with inflammation were it not that it is frequently acted on by morbid-excitants, the influence of which seldom extends beyond the alveolar border and face. Febrile and gastric affections, eruptic diseases, as measles, and small-pox, syphilis, excessive use of mercurial medicines, scorbutic or scrofulous diathesis of the general system, and anything that will enervate the vital powers of the body, increases its irritability.

Boyer describes the symptoms as a very severe fixt and deep-seated pain under the cheek, extending from the alveolar border to the lower part of the orbit, local heat, pulsation and sometimes fever. Deschamps distinguishes the symptoms from other affections of the cavity by a dull, heavy pain in the region of it, which, he says, becomes sharp, and extends from the alveolar to the frontal sinus. He tells us this malady cannot be confounded with any other, if there is no external visible cause. Suppuration and ulceration have peculiar signs which cannot be confounded with those of inflammation.

### IN PRACTICE.

In the latter part of the summer of 1862, A. Anderson, a potter by trade, residing in our town (Delphi), was attacked by a severe pain in the antrum, extending, as Boyer describes it, from the alveolar border to the orbit. He applied to his physician, Dr. Thomas, who thought that the extraction of the first superior left molar would relieve him; but, in attempting it, failed. He then sent the patient to me, but I failed. The pain grew so severe as to almost distract him. I advised him to go to Lafayette and consult Dr. Moore, and, at his request, accompanied him. Dr. Moore tried to extract it, but failed. He then extracted the wisdom tooth, thinking it would relieve him. It was with difficulty he extracted it, and no relief was given. He then perforated the antrum with a drill; but no pus followed. The pain ceased and we returned home. I kept a pledget of cotton dipt in creosote in the orifice for several weeks, renewing it daily. In the meantime I had occasion to go east, and, in my absence, he neglected it. When I returned I found the orifice closed, and the pain as severe as at first. I perforated it again, and applied the cotton pellets with creosote, and, continuing it for six or eight weeks, succeeded in effecting a cure. He had no decayed teeth in either jaw.

In the spring of 1871 Dr. John Richardssn (now dead), of Delphi, had a scrofulous case that baffled him. A young man of eighteen years was attacked with a severe pain in the frontal sinus. The doctor brought him to my office to have me examine his teeth. I found them but little decayed. After a consultation we agreed, the pain being on the right side, to perforate the sinus near the front and lateral incisors in two places, which I did applying carbolic acid on a pellet of cotton in the orifices, renewed it every day. This I continued for about two months, the doctor at the same time treating him.

The pain ceased, but the doctor continued his treatment till his patient fully recovered. He is at this time in good health, and while his teeth are not in the best condition, the roots are filled, and he has not experienced pain in the sinus since I treated it.

A minister of the gospel in Delphi having the superior left lateral incisor decayed wished to have it filled. The nerve being dead, I proceeded to treat preparatory to filling the fang and tooth. By the time I was ready to fill it, he accidently broke it off. I then filled the extremity of the fang, leaving it for some time before inserting a tooth, to see if it was properly filled. I then inserted a Bonwill tooth. After wearing it some time he broke it. I put on another, and after wearing it for some time an abscess formed near the front tooth. I attempted to heal it and failed. I then advised him to have it extracted, but he refused. I continued treating it, but had no success in getting it healed. I at length succeeded in extracting it, but found no inflammation in the periosteum. I treated with carbolic acid for some time, and it seemed to grow worse instead of better. A physician who knew of the case, looked at the abscess, and, probing it, found the process around the front incisor exfoliated. I then commenced cutting the exfoliation away, syringing once a day with listerine. Some ten or more years ago he had the lachrymal ducts operated on, since which matter would form in the corner of the eye, and the tears would flow over instead of through the duct. The abscess appeared to follow to the apex of the tooth directly toward the duct. The disease has been there for a great while, and is not altogether healed at this date.—*Ohio State Journal.*

